# Serum TSH, 25(OH) D and phosphorus levels predict weight loss in individuals with diabetes/prediabetes and morbid obesity: a single-center retrospective cohort analysis

**DOI:** 10.1186/s12902-022-01202-4

**Published:** 2022-11-18

**Authors:** Kemal Ağbaht, Serhan Vahit Pişkinpaşa

**Affiliations:** 1 Defne Hospital, Endocrinology and Metabolic Diseases Department, Odabaşı Mahallesi, Uğur Mumcu Bulvarı, No: 101, Antakya, Hatay Turkey; 2 Iskenderun State Hospital, Nephrology Department, İskenderun, Hatay Turkey

**Keywords:** Morbid obesity, Type 2 diabetes, Thyroid stimulating hormone, Vitamin D, Phosphorus

## Abstract

**Background:**

To evaluate the association of vitamin D and thyroid-stimulating hormone (TSH) with weight loss (WL) percentage (%) in patients with diabetes/prediabetes and Class II/III obesity.

**Methods:**

A retrospective cohort study was designed. Data were collected from a database of a referral endocrinology clinic that is prospectively and systematically generated. After exclusion of unavailable cases, the study enrolled 285 patients (51 ± 11 years old, female/male = 208/77; diabetes/prediabetes = 159/126; no/on levothyroxine replacement = 176/109; Class II/III obesity = 184/101, respectively) who maintained euthyroidism and were followed up for ≥6 months. The data were analyzed to determine the predictors of WL%.

**Results:**

Compared with baseline, in the median 22 months of follow-up, the whole study group lost 5.1% of their baseline body weight. As most obesity management trials define success as ‘at least 10% of WL compared to baseline’, we stratified the patients based on WL% extents. The distribution was as follow: Group 1 (*n* = 61) lost ≥10% body weight, Group 2 (*n* = 162) lost < 10% body weight, while Group 3 (*n* = 62) gained weight by the final visit. In groups 1 and 2 (weight losers), the serum thyroid stimulatig hormone (TSH) and parathyroid hormone (PTH) levels decreased and the free thyroxine (fT4), calcium, phosphorus, and 25-hydroxyvitamin D (25(OH)D) levels increased. In Group 3 (weight gainers), these changes were not observed (except for an increase in calcium levels). Regression analysis revealed that the final visit TSH (β = − 0.14, *p* < 0.05), 25(OH) D (β = 0.15, *p* < 0.05), and phosphorus (β = 0.20, *p* < 0.05) levels predicted WL%. However, if patients with autoimmune thyroiditis were excluded from the analysis, the decrease in TSH levels was not statistically significant.

**Conclusions:**

Serum TSH, phosphorus, and 25(OH) D levels predict WL% in euthyroid patients with diabetes/prediabetes and morbid obesity. TSH predictivity seems to be a function of thyroid autoimmunity present with increased frequency in this cohort. Greater levels of phosphorus within the reference range and a sufficient vitamin D status are associated with a greater WL%.

**Supplementary Information:**

The online version contains supplementary material available at 10.1186/s12902-022-01202-4.

## Background

Obesity is a chronic disease that has significant adverse health outcomes and is responsible for most cases of type 2 diabetes (T2DM), hypertension, dyslipidemia, and an increased risk of cardiovascular disease [1]. As the severity of obesity increases, the possibility of developing serious health complications also increases. For example, the risk of glucose metabolism impairment and T2DM is substantially increased in patients with Class II and Class III obesity, and due to these comorbidities, these patients are considered morbidly obese [2].

One of the major challenges in the long-term medical management of T2DM and prediabetes is to achieve sustainable weight loss (WL) [3]. Currently, pharmacological approaches that may help achieve this goal are scarce and usually require a combination of drugs [4–6]. This inadequacy of medications to achieve efficient WL is attributed to the complexity of diseases that are caused by a combination of genetic, environmental, and lifestyle factors [7]. However, there is an increased prevalence of concomitant hormonal diseases, such as hypothyroidism and severe vitamin D deficiency, which occur in obese individuals and may complicate WL. These deficiencies must be properly diagnosed and managed [8].

The current guidelines recommend testing all patients with obesity for thyroid function, given the high prevalence of hypothyroidism in obesity. However, due to the very low quality of the evidence on the benefit of vitamin D supplementation, it is not recommended to test these patients for vitamin D deficiency or hyperparathyroidism [8]. The previously published guidelines recommended screening individuals at risk for vitamin D deficiency, for which obesity is a known risk factor [9]. Decreased 25-hydroxyvitamin D (25(OH)D) bioavailability due to body fat sequestration, malnutrition (low vitamin D intake), sun avoidance, decreased levels of 25(OH)D-binding protein, and decreased liver synthesis contribute to the lower 25(OH) D levels observed in obese patients [8]. In accordance with these observations, data from a previous cross-sectional study showed an inverse relationship between obesity and circulating 25(OH) D levels and calcium-phosphorus product [10]. One of the well-known actions of the active (hormonal) form of 25(OH) D is phosphate absorption in the intestine [11]. Suppressing elevated parathormone increases the reabsorption of phosphate from the proximal tubules of the kidney, thereby preventing urinary wasting of phosphorous [12]. Due to its widespread distribution and important role in vital cellular processes such as adenosine triphosphate production (ATP), insufficient phosphorus levels may promote muscle weakness, impaired leukocyte function [13], and obesity [14].

In the present study, we aimed to document the association of WL% with thyroid hormones, their calculated derivatives, 25(OH)D, parathyroid hormone (PTH), calcium, and phosphorus levels in a cohort of patients with T2DM or prediabetes and Class II or Class III obesity at baseline and to address the probable mechanisms involved in these associations. The study was conducted in an outpatient referral Endocrinology Clinic, in a tertiary care facility hospital located in Hatay, a city located in the south of Turkey, with a high prevalence of both obesity and T2DM. The present study analyzed data of 5.3 years, with a median follow-up duration of 22 months. To our knowledge, this is the first study to thoroughly assess these associations in this population. Most confounding factors were eliminated while enrolling the cohort.

## Methods

### Study population and design

Data from a systematically generated database from our adult outpatient endocrinology clinic (Defne Hospital, Hatay, Turkey) was used in this retrospective cohort study. During a consultation by a professional nurse, all patients provided information regarding any history of chronic disease, smoking and alcohol habits, medication history, the specific reason for the clinic visit, a family history of diabetes, and any other symptoms they were experiencing. Anthropometric measurements were taken and body composition analysis was performed after 10–12 h of fasting, using a Tanita-type BC-418 body composition analyzer (Tanita Corporation, Tokyo, Japan). Blood pressure was also measured. Subsequently, the patient was examined by a clinical endocrinologist. The patients’ medical histories, medications, symptoms, and physical examination details were noted. For patients who had a body mass index (BMI) ≥35 kg/m^2^, a series of blood tests were ordered. These blood tests measured the level of serum fasting glucose, insulin, hemoglobin A1c (HbA1c), 25(OH)D, parathormone, albumin, thyroid hormones, electrolytes, liver and kidney function, lipid profiles, and a complete blood count. Other tests were conducted if necessary. Depending on the test results, the patients were prescribed appropriate medications, offered a consultation to a dietitian for medical nutrition therapy, and regular physical activity was encouraged. Patients with 25(OH)D levels < 50 nmol/L were given a supplementation [9]. Throughout the study, these data were recorded in the database.

The data from patients with a BMI ≥35 kg/m^2^ who had visited the clinic at least twice between December 2014 and March 2020 were included in this study. Exclusion criteria are provided in Supplementary Table 1.

For the patients who met the inclusion criteria, information such as their medical history, anthropometric measurements, body fat analysis, medication usage, and thyroid stimulating hormone (TSH), free thyroxine (fT4), free triiodothyronine (fT3), 25(OH)D, calcium, phosphorus, albumin, and PTH levels in the blood, were documented. The calculated sum activity of step-up deiodinases (SPINA-GD) and the calculated thyroid’s secretory capacity (SPINA-GT) indices were calculated using the SPINA Thyr (research version) software. Corrected calcium was derived from the measured calcium using the following equation: (albumin in g/L – 40) × 0.08, and the corrected calcium value was used in all analyses. Blood test results and other notable parameters were analyzed in relation to the weight change between the first and last visits.

After documenting the blood test results obtained at both the baseline and follow-up visits, their associations with weight change were analyzed in detail.

#### Definitions

##### Type 2 diabetes mellitus

Fasting plasma glucose ≥7.0 mmol/L (≥ 126 mg/dL), or 2-hour postprandial glucose ≥11.1 mmol/L (≥ 200 mg/dL), or HbA1c ≥ 6.5% (≥ 48 mmol/mol) [15].

##### Prediabetes

Fasting plasma glucose 5.6–6.9 mmol/L (≥ 100–125 mg/dL), or 2-hour postprandial glucose 7.8–11.0 mmol/L (140–199 mg/dL), and/or HbA1c between 5.7–6.4% (39–47 mmol/mol) [15].

##### Spina-GD

Refers to the deiodination capacity (total deiodinase activity). It tests the maximum amount of T3 produced from T4 by peripheral deiodinases (reference range 20–40 nmol/s) [16].

##### Spina-GT

Refers to thyroid hormone output (thyroid’s incretory capacity). It tests the maximum amount of T4 produced by the thyroid in 1 s (reference range 1.41–8.67 pmol/s). It is not available for subjects who are undergoing levothyroxine (LT4) replacement therapy [16].

Other definitions, hormone assays and other measurements are provided in Supplementary Table 2.

### Statistical analysis

Continuous variables were expressed as the mean ± standard deviation or median and interquartile range [median (IQR25–IQR75)]. Categorical variables were compared using Pearson’s chi-square test. Changes in continuous variables within the same groups (such as BMI and TSH) between the first and last visits were compared using the Wilcoxon signed rank test. When the patients were categorized into three or more groups and compared for several parameters, the changes in continuous variables between groups were compared using one-way ANOVA or the Kruskal Wallis test. Two groups were compared using the Mann-Whitney U test.

Since the study was a retrospective cohort analysis and identified all the patients that were admitted during the 5.3-year study period, power analysis was not calculated.

WL% was calculated as ‘1-(weight at the last visit/weight at the first visit)’.

To determine possible associations between weight change and the blood test results, as well as between other variables, a linear regression analysis, enter method was performed, as weight change was the dependent variable. Variables that have a plausible association with weight change and those that were found to be probably associated in the univariate analysis, were included in the models as independent variables. Linear model assumptions that are linearity and additivity of the relationship between dependent and independent variables, statistical independence of the errors, constant variance of the errors, and normality of the error distribution, were tested across all models. The terms used in the analysis are described in Supplementary Table 2.

A cox proportional hazard regression model was used to assess weight loss in the investigated subgroups over time.

Partial correlation was performed to adjust for the effects of other probable confounding factors when analyzing the correlation of WL% with the classification based on 25(OH)D and phosphorus level theories.

SPSS (version 22.0; IBM Corp, Armonk, NY, USA.) was used for all statistical calculations.

## Results

### Patient characteristics

**Fig. 1:**
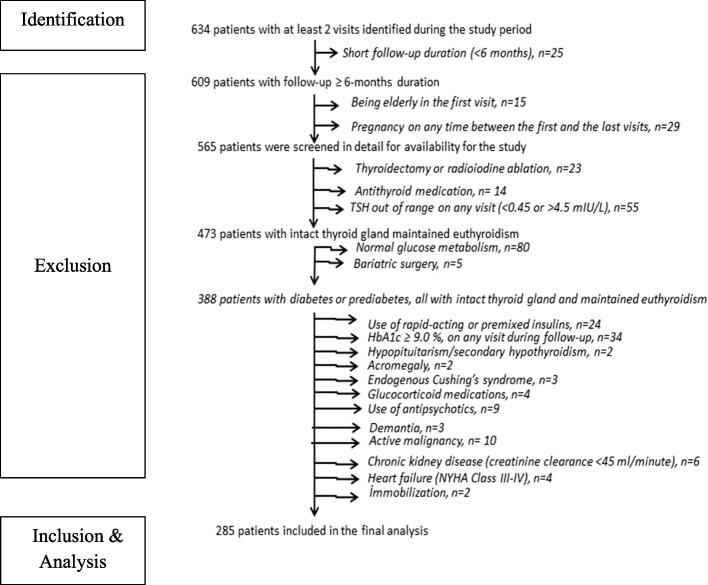
Flow diagram of the study

During the study period, 634 patients with a BMI ≥35 kg/m^2^ visited our endocrinology clinic two or more times. Patients with a short follow-up duration (< 6-months, *n* = 25), those older than 75 years of age (*n* = 15), and those who were pregnant (*n* = 29) were excluded from the study. Further investigations led to the exclusion of an additional 280 patients due to a variety of reasons that may confound weight management or due to thyroid dysfunction, normal glucose metabolism, or bariatric surgery. Analysis was performed on the remaining 285 patients. A flow diagram of the study is shown in Fig. 1.

The mean age of the study population was 51.3 ± 11.1 years old, with a female predominance (*n* = 208, 73.0%), of whom 120 (57.7%) were postmenopausal. The median follow-up duration was 22 months. Most patients had T2DM (*n* = 159, 55.8%), and the remaining patients had well managed prediabetes (*n* = 126, 44.2%). Overall, 109 patients (38.2%) were receiving levothyroxine replacement; and 64 (22.5%) had circulating thyroid autoantibodies, of whom 43 (67.2%) had received levothyroxine replacement and 21 (32.8%) had not. The majority (64.6%, *n* = 184) of the patients had Class II obesity, and the remaining (35.4%, *n* = 101) had Class III obesity. The last visit was in winter for 77 (27.0%), in spring for 66 (23.2%), in summer for 54 (18.9%), and in fall for the remaining 88 (30.9%) of the patients. Other patient characteristics are detailed in Supplementary Table 3. The drugs frequently used by the patient cohort are detailed in Supplementary Table 4.

### Anthropometric and blood test measurements

These changed during the study period, with a median body weight of 103 kg and a BMI of 38.2 kg/m^2^ at the first visit. Both men (median 35.9%) and women (median 46.0%) had a high body fat percentage (BFP) at the beginning of the study. The study population lost substantial weight and fat in the follow-up period (weight at the last visit was 97 kg, and BMI was 36.6 kg/m^2^, *p* < 0.001, for both). WL% and total body fat loss percentage were strongly correlated in men (r = 0.93, p < 0.001) and women (r = 0.90, p < 0.001). There was no difference, at the beginning or at the end of the study, in final weight, BMI, or BFP between the group of patients who received levothyroxine replacement and those who did not (Table 1). At the beginning of the study, there was a noticeable difference in the serum TSH and fT4 levels between these two groups; however, by the end of the study, the difference in TSH levels was no longer apparent, indicating the successful maintenance of euthyroidism by levothyroxine replacement during the follow-up period (Table 1).

**Table 1:** Comparison of anthropometric and blood test measurements in individuals receiving or not receiving levothyroxine replacement during the first and last visit

	No Levothyroxine (LT4) replacement, ***n*** = 176			On Levothyroxine (LT4) replacement, ***n*** = 109			*p*-value, between the groups	
	First visit	Last visit	*p*-value*	First visit	Last visit	*p*-value*	First visits**	Last visits**
Weight, kg	**104 (94–118)**	**98 (91–112)**	**<.001**	**101 (94–118)**	**97 (89–110)**	**<.001**	.731	.566
BMI, kg/m^2^	**37.8 (36.0–42.1)**	**36.5 (35.1–39.4)**	**<.001**	**38.7 (36.2–43.3)**	**37.2 (34.8–40.9)**	**<.001**	.198	.377
BFP (%), men	**35.6 (32.1–38.0)**	**33.4 (30.5–36.0)**	**<.001**	**38.0(34.2–43.3)**	**35.9 (32.0–41.4)**	**.022**	.111	.221
BFP (%), women	**45.7 (43.7–48.9)**	**44.4 (42.6–47.0)**	**<.001**	**46.2 (42.8–49.2)**	**44.7 (42.0–47.4)**	**<.001**	.688	.597
TSH (μIU/L)	1.2 (0.9–1.6)	1.2 (0.9–1.6)	.841	**2.2 (1.6–2.7)**	**1.3 (0.9–2.0)**	**<.001**	**<.001**	.113
fT4 (pmol/L)	13.3 (12.4–14.5)	13.4 (12.4–14.7)	.520	**12.7 (11.6–13.8)**	**14.0 (12.8–15.1)**	**<.001**	**.001**	**.017**
fT3 (pmol/L)	**4.3 (3.9–4.7)**	**4.3 (4.0–4.9)**	**.020**	4.2 (3.8–4.8)	4.2 (3.9–4.6)	.412	.550	**.083**
SPINA-GD (nmol/s)	**30 (26–33)**	**30 (27–35)**	**.012**	**31 (26–35)**	**28 (24–32)**	**<.001**	.183	**.004**
SPINA-GT (pmol/s)	3.6 (2.8–4.3)	3.4 (2.7–4.4)	.419	–	–	–	–	–
Calcium (mg/dL)	**9.2 (8.9–9.4)**	**9.6 (9.3–9.8)**	**<.001**	**9.1 (8.8–9.4)**	**9.5 (9.1–9.7)**	**<.001**	.114	.094
Phosphorus (mg/dL)	**3.3 (3.1–3.7)**	**3.5 (3.2–3.8)**	**<.001**	3.5 (3.1–3.8)	3.6 (3.1–3.8)	.066	.133	.497
Parathormone (pg/mL)	**72 (56–103)**	**67 (48–89)**	**.001**	67 (49–91)	**65 (52–83)**	**.045**	.427	.984
25(OH)D (nmol/L)	**36 (24–48)**	**53 (41–69)**	**<.001**	**31 (21–49)**	**56 (40–69)**	**<.001**	.266	.705

*BMI* body mass index, *BFP* body fat percentage, *fT3* free triiodothyronine, *fT4* free thyroxine, *SPINA-GD* sum activity of step-up deiodinases, *SPINA-GT* the calculated thyroid’s secretory capacity

*Comparisons were performed by Wilcoxon signed rank test

**Comparisons were performed by Kruskal-Wallis test

### The extent of WL% and its association with thyroid hormones, 25(OH)D, and the related measurements

Overall, the study population lost 5.1% of its baseline weight. As most obesity management trials define success of a particular management as achieving 10% of weight loss, we chose this cutoff value when constructing the groups for comparisons. Patients were stratified based on the extent of their WL%: Group 1 lost ≥10% of their baseline weight (*n* = 61, 21.4%), Group 2 lost < 10% of their baseline weight (*n* = 162, 56.8%), [when this subgroup was further divided, 26.3% (*n* = 75) had lost 5–9.9%, and the remaining 30.5% (*n* = 87) had lost < 5%], and Group 3 gained weight (*n* = 62, 21.8%). The serum TSH levels decreased from 1.3 to 1.2 μIU/L in Group 1, and from 1.5 to 1.3 μIU/L in Group 2. Similarly, PTH levels decreased, while fT4, 25(OH)D, and phosphorus levels increased in both groups. None of these changes were observed in Group 3. The serum calcium levels increased and the serum fT3, SPINA-GD, and SPINA-GT indices remained constant in all three groups (Table 2).

**Table 2:** Comparison of blood test measurements of individuals categorized according to the extent of their weight loss

	Weight loss ≥10%, (*n* = 61)			Weight loss < 10%, (*n* = 162)			Weight gain, (*n* = 62)			p-value **	p-value **
	First visit	Last visit	*p*-value*	First visit	Last visit	*p*-value*	First visit	Last visit	*p*-value*	*between groups, first visit*	*Between groups, last visit*
TSH (μIU/L)	**1.3 (1.0–2.1)**	**1.2 (0.8–1.5)**	**<.001**	**1.5 (1.1–2.2)**	**1.3 (0.9–1.7)**	**<.001**	1.6 (1.1–2.1)	1.4 (1.1–2.0)	.058	.346	**.009**
fT4 (pmol/L)	**13.2 (12.3–14.5)**	**13.9 (12.6–15.0)**	**.018**	**13.1 (12.2–14.2)**	**13.6 (12.4–14.7)**	**.042**	12.7 (11.6–14.1)	13.3 (12.2–14.7)	.112	.357	.225
fT3 (pmol/L)	4.2 (3.6–4.7)	4.1 (3.8–4.7)	.422	4.2 (3.9–4.7)	4.3 (4.0–4.8)	.089	4.4 (4.1–4.8)	4.3 (4.0–4.8)	.907	.069	.347
SPINA-GD (nmol/s)	28 (23–33)	28 (24–32)	.755	30 (26–33)	30 (26–34)	.770	31 (28–37)	30 (26–34)	.221	.058	.137
SPINA-GT (pmol/s)	3.7 (2.8–4.3)	3.6 (3.1–4.6)	.894	3.2 (2.5–4.0)	3.3 (2.6–4.3)	.443	3.0 (2.2–3.9)	3.3 (2.3–3.8)	.904	.145	.059
Calcium (mg/dL)	**9.2 (9.0–9.4)**	**9.6 (9.3–10.0)**	**<.001**	**9.1 (8.9–9.5)**	**9.5 (9.2–9.7)**	**<.001**	**9.1 (8.7–9.4)**	**9.5 (9.1–9.8)**	**<.001**	.559	.058
Phosphorus (mg/dL)	**3.5 (3.0–3.7)**	**3.7 (3.4–4.1)**	**<.001**	**3.4 (3.1–3.7)**	**3.5 (3.2–3.9)**	**<.001**	3.4 (3.1–3.6)	3.3 (3.1–3.7)	.480	.877	**.002**
Parathormone (pg/mL)	**72 (59–92)**	**62 (49–74)**	**.001**	**75 (51–99)**	**64 (48–82)**	**<.001**	66 (51–98)	79 (61–95)	.572	.803	**.004**
25(OH)D (nmol/L)	**31 (19–45)**	**64 (46–78)**	**<.001**	**34 (22–48)**	**55 (41–69)**	**<.001**	40 (23–54)	47 (30–56)	.174	.278	**<.001**
Follow-up duration (months)	22 (13–43)			20 (11–33)			24 (14–34)			.145**	
Levothyroxine replacement, n = 109 (%)	24 (39.3)			65 (40.1)			20 (32.3)			.545***	
Anti-thyroid antibody positivity, *n* = 64 (%)	13 (21.3)			33 (20.4)			18 (29.0)			.370***	

*Comparisons were performed by Wilcoxon signed rank test

**Comparisons were performed by Kruskal-Wallis test

***Comparisons were performed by Pearson’s Chi-square

When the nine blood test measurements (TSH, fT4, fT3, SPINA-GD, SPINA-GT, 25(OH)D, PTH, calcium and phosphorus), were compared between the three groups, no differences at baseline were observed. At the end of the study, the TSH and PTH levels had decreased (Group 1 < Group 2 < Group 3), while phosphorus and 25(OH)D increased (Group 1 > Group 2 > Group 3) (Table 2, the last two columns). The follow-up duration, the proportion of anti-thyroid antibody positivity, and levothyroxine replacement were similar among the three groups (Table 2).

### Correlations of WL% with thyroid hormones, 25(OH)D, and the related measurements

Spearman’s correlation analysis revealed a positive correlation between the final visit serum calcium, phosphorus, and 25(OH)D levels with WL%, while TSH and PTH levels were negatively correlated with WL in the entire study population (Supplementary Table 5). When the correlation analysis was restricted to patients who did not receive levothyroxine replacement, the change in PTH levels was not statistically significant; however, there was a correlation between SPINA-GT and WL (Supplementary Table 5).

Several regression analysis models were performed on the entire study population as well as on a restricted group of patients who did not receive levothyroxine replacement. This was done to exclude the probable effects of confounding factors or severity of multicollinearity (Table 3). The models showed that greater cumulative doses of metformin, orlistat, a greater baseline BMI, higher levels of final visit serum 25(OH)D and phosphorus, and lower levels of TSH had a significant association with WL%, but levothyroxine treatment revealed no independent association with WL%. If patients with thyroid autoantibody positivity were excluded, TSH levels did not have a statistically significant association with WL% (Table 3, Model 3). Models 1, 2, and 3, which were restricted to patients who did not receive levothyroxine treatment, indicated that approximately half (R^2^ > 0.50) of the total variation in weight change was explained by the variance of these variables. When the entire group was considered, this value was 0.43. The VIF for collinearity was < 5 for all variables (Table 3).

**Table 3:** Regression analysis of weight loss in the study population

	Model 1, no LT4 replacement group (*n* = 176)			Model 2, no LT4 replacement group (*n* = 176)			Model 3, anti-thyroid antibody negative and no LT4 replacement group (*n* = 155)			Model 4, The entire group (*n* = 285)		
	***R2 = 0.52, F = 10.1, p < .001***			***R2 = 0.54, F = 11.5, p < .001***			***R2 = 0.51, F = 9.1, p < .001***			***R2 = 0.43, F = 13.4, p < .001***		
	Beta	*p*-value	VIF	Beta	*p*-value	VIF	Beta	*p*-value	VIF	Beta	*p*-value	VIF
TSH, last visit	**−0.14**	**.046**	1.20	–	**–**	**–**	− 0.13	.081	1.18	**− 0.15**	**.006**	1.11
fT4, last visit	− 0.01	.926	1.13	–	–	–	− 0.01	.170	1.11	0.03	.532	1.12
fT3, last visit	−0.04	.541	1.08	–	–	–	− 0.00	.986	1.06	−0.06	.271	1.10
SPINA-GD, last visit	–	–	–	0.01	.870	1.23	–	–	–	–	–	–
SPINA-GT, last visit	–	–	–	0.09	.167	1.16	–	–	–	–	–	–
Calcium, last visit	0.08	.203	1.18	0.08	.257	1.20	–	–	–	–	–	–
Phosphorus, last visit	**0.18**	**.008**	1.27	**0.16**	**.021**	1.30	**0.20**	**.006**	1.21	**0.13**	**.019**	1.21
25(OH)vit D, last visit	**0.14**	**.032**	1.11	**0.13**	**.050**	1.12	**0.16**	**.022**	1.11	**0.16**	**.002**	1.09
Parathormone, last visit	0.00	.998	1.18	0.02	.973	1.20	0.02	.825	1.24	−0.09	.095	1.18
BMI, at baseline	**0.36**	**<.001**	1.77	**0.39**	**<.001**	1.67	**0.39**	**<.001**	1.85	**0.34**	**<.001**	1.52
Orlistat,^a^	**0.21**	**.016**	1.94	**0.21**	**.011**	1.84	**0.19**	**.050**	2.01	**0.19**	**.003**	1.56
Metformin^a^,	**0.20**	**.011**	1.58	**0.21**	**.004**	1.49	**0.20**	**.017**	1.61	**0.15**	**.015**	1.45
Acarbose^a^	0.04	.559	1.49	0.07	.356	1.44	0.08	.308	1.8	0.10	.089	1.32
Exenatide, ^a^	0.04	.587	1.56	0.02	.821	1.59	0.02	.772	1.63	0.08	.232	1.58
SGLT-2 inh. ^a^	−0.03	.693	1.55	0.00	.996	1.55	−0.05	.511	1.55	−0.02	.710	1.58
Fluoxetine^a^	0.04	.618	1.47	0.03	.725	1.50	0.00	.959	1.58	0.02	.768	1.37

^a^Cumulative doses

### Serum 25(OH)D and phosphorus levels and prediction of WL%

**Fig. 2:**
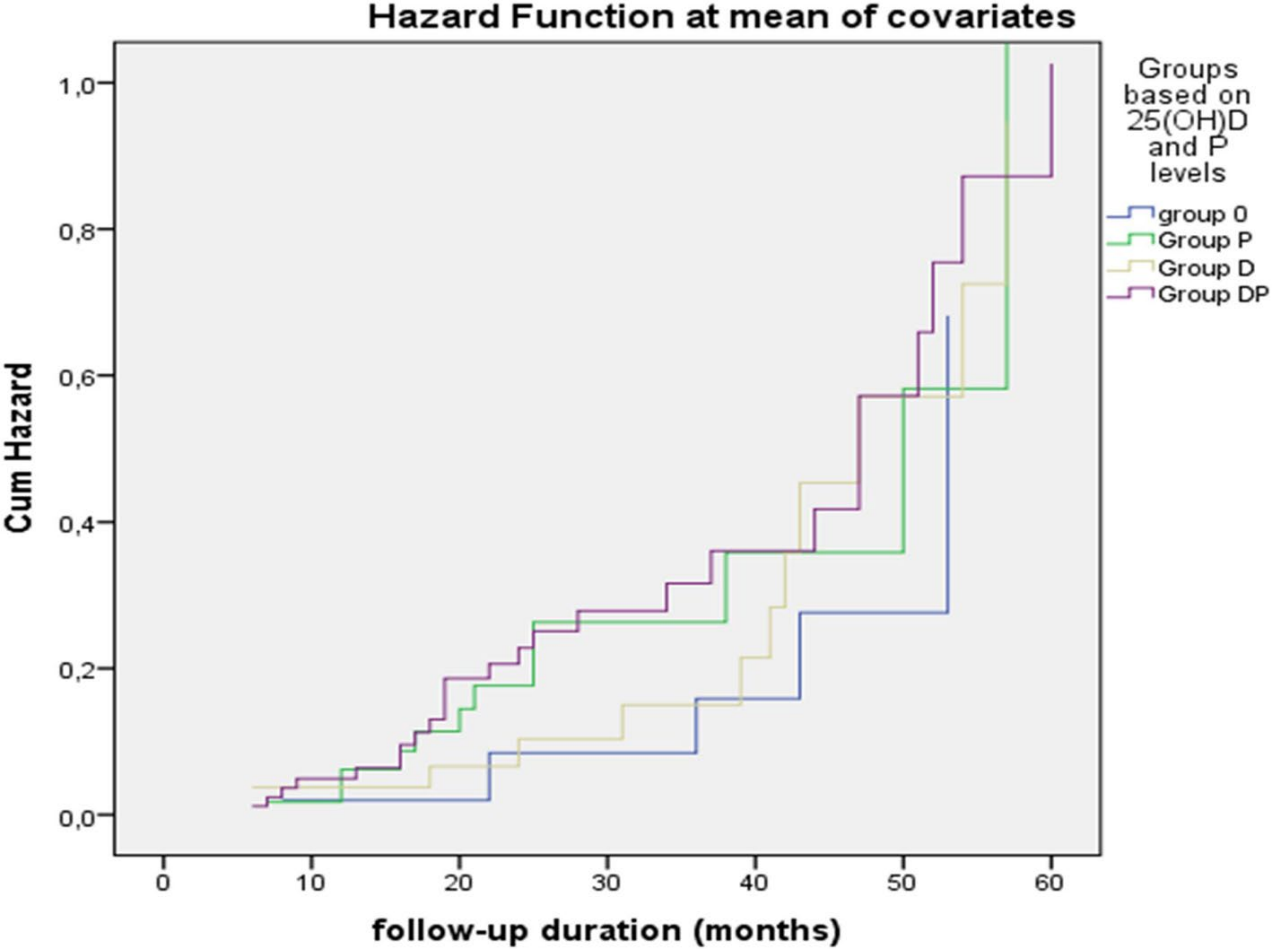
A cox proportional hazard regression model comparing group DP, group D, group P, and group 0 for 10% loss of baseline weight during follow-up

A simple scale was established to determine the levels measured at the last visit of 25(OH)D (below or above 50 nmol/L), and phosphorus (below or above 3.5 mg/dL) in the patients. The patients were broken down into groups according to 25(OH)D and phosphorus levels as follows: group DP included patients with sufficient 25(OH)D [25(OH)D ≥ 50 nmol/L] (9), and higher phosphorus levels (upper half of the reference range: 3.5–4.5 mg/dL), group D included patients with sufficient 25(OH)D, but lower phosphorus levels (lower half of the reference range: 2.5–3.49 mg/dl), group P included patients with higher phosphorus but insufficient 25(OH)D levels, and Group 0 included patients with low levels of 25(OH)D and phosphorus (Table 4). A one-way ANOVA was performed to compare the effect of these four groups based on 25(OH)D and phosphorus levels (group DP, group D, group P, group 0) on weight loss and fat mass loss. Although the BMI was similar at baseline in the four groups, a one-way ANOVA revealed that there was a statistically significant difference in mean weight (F: 8.9, *p* < 0.001) and fat mass losses (F: 8.6, *p* < 0.001) between at least two groups. Bonferonni test for multiple comparisons found that the mean value of weight loss was significantly different between group 0 and the other three groups (group DP, *p* < 0.001, group D, *p* = 0.019, group P, *p* = 0.027). On the other hand, there was no statistically significant difference in mean weight loss between the other three groups (group DP and group P, *p* = 0.217, or between group DP and group D, *p* = 0.392, or between group D and group P, *p* = 0.999). A cox proportional hazard regression model demonstrated that during follow-up group DP had the highest, and group 0 had the lowest hazard ratio to lose 10% of baseline weight (Fig. 2). The analysis for fat mass yielded similar results (Table 4). Group DP was further divided to separate patients who had 25(OH)D levels ≥75 nmol/L and phosphorus levels ≥4.0 mg/dL (*n* = 11). When analyzed separately, this subgroup lost 10.0% (2.3–16.5) of weight and 15.4% (6.2–30.5) of fat mass.

**Table 4:** Comparison of criteria based on 25(OH)D and phosphorus levels in the last visit *

	Group DP [25(OH)D ≥ 50 nmol/L, Pi≥3.5 mg/dL], (*n* = 87)	Group D [25(OH)D ≥ 50 nmol/L, Pi < 3.5 mg/dL] (*n* = 54)	Group P [25(OH)D < 50 nmol/L, Pi≥3.5 mg/dL] (*n* = 59)	Group 0 [25(OH)D < 50 nmol/L, Pi < 3.5 mg/dL] (*n* = 55)	*p*-value, for the trend
BMI at baseline (kg/m^2^)	37.8 (36.0–42.1)	37.3 (35.9–40.8)	38.9 (36.4–45.2)	38.0 (35.9–40.5)	.222**
BMI in the final visit (kg/m^2^)	36.0 (34.3–38.8)	35.9 (34.8–38.6)	37.8 (35.7 (41.9)	37.4 (35.6–40.4)	.002**
Weight loss (%), (95% Confidence interval)	6.3 (3.1–9.6)	4.1 (0.4–7.7)	3.8 (0.3–7.3)	Reference	a,b***
Fat mass loss (%), (95% Confidence interval)	11.1 (5.2–17.0)	8.1 (1.5–14.6)	7.3 (1.0–13.7)	Reference	a,b***
Weight loss ≥10%, (*n* = 61)*	26 (29.9)	12 (22.2)	12 (20.3)	6 (10.9)	<.001****
Weight loss 5–9.9%, (*n* = 75)*	28 (32.2)	13 (24.1)	13 (22.0)	11 (20.0)	
Weight loss < 5%, (*n* = 87) *	23 (26.4)	17 (31.5)	21 (35.6)	15 (27.3)	
Weight gain (*n* = 62)*	10 (11.5)	12 (22.2)	13 (22.0)	23 (41.8)	

*There were 30 missing values

**Comparisons were performed by Kruskal-Wallis test

***Comparisons were performed by one-way ANOVA

a: *p* < 0.001 (comparison between DP and 0)

b: *p* < 0.05 (comparison between D and 0, comparison between P and 0)

****Comparisons were performed by Pearson’s Chi-square

This classification was further simplified, group DP was assigned 2 points, group D and group P were assigned 1 point, and group 0 was assigned 0 points, based on the criteria that satisfied the sufficiency. The correlation of WL% with this simple classification was consistent after adjusting for age, sex, the season which the last visit was done, menopausal status, baseline BMI, follow-up duration, anti-thyroid antibody positivity, the presence of diabetes or prediabetes, serum calcium levels, cumulative doses of levothyroxine, metformin, orlistat, acarbose, sodium-glucose cotransporter-2 inhibitors, exenatide, basal insulin, insulin secretagogues, diuretics, beta-blockers, and fluoxetine medications (*r* = 0.31, *p* < 0.001).

## Discussion

The novelty of the present study is the detailed evaluation of probable predictors of WL%, including serum TSH, fT4, fT3, the calculated total deiodinase, thyroid’s incretory capacities, 25(OH)D, PTH, calcium, and phosphorus levels in patients with morbid obesity. All the study participants maintained TSH, phosphorus, and calcium levels within the reference ranges during the follow-up period. Although serum calcium levels increased in all the subgroups, only an increased serum phosphorus and 25(OH)D level, and decreased serum TSH level independently predicted the extent of WL% in this study group. More prominent WL was observed in patients who had sufficient levels of both 25(OH)D (≥ 50 nmol/L) and phosphorus (defined as levels in the upper half of the reference range).

In a population-based study, after the exclusion of subjects with previous or present overt thyroid dysfunction, it was shown that thyroid function within the normal range could be one of the many factors that act in concert to determine body weight. Even a slightly elevated serum TSH level is associated with an increased risk of obesity [17]. Similarly, a positive correlation was observed between increased serum leptin and TSH levels in euthyroid obese women [18]; the increased amount of fat in patients with severe obesity could result in an increase in TSH and leptin levels [19]. Marzullo et al. (2010) observed an increased risk of hypothyroidism in morbidly obese individuals. This may be due to the immunoregulatory effects of leptin, which could eventually result in the development of an autoimmune thyroid disease [20]. These findings suggest that obese patients are more prone to develop autoimmune hypothyroidism, and even mild hypothyroidism, which contributes to the progressive increase in body fat mass, may ultimately result in obesity. Another study reported that obese individuals often have a slightly elevated level of serum TSH, which may not be indicative of thyroid hormone failure. Therefore, elevated TSH levels are a consequence, rather than the cause of excess weight [21]. In our study, the prevalence of autoimmune thyroid disease was 22.5%, of which approximately two-thirds of these patients had received levothyroxine replacement. In contrast, a greater proportion (38.2%) of the entire study population had received levothyroxine replacement; therefore, only 39.4% of patients receiving thyroid hormone replacement were anti-thyroid antibody-positive. The remaining patients may have received levothyroxine replacement due to mildly elevated TSH levels. This is inferred because patients with thyroidectomy, hypopituitarism, and other causes of thyroid dysfunction were excluded from the study. Despite thyroid hormone replacement, WL% did not differ between patients who had and who had not received levothyroxine replacement. If the entire study population was considered, the TSH level independently predicted the extent of WL%. However, if the analysis was confined to patients who were negative for thyroid autoantibodies and did not undergo thyroid hormone replacement, the TSH level was no longer a significant predictor of WL%. In the univariate analysis, thyroid’s incretory capacity was associated with WL% in patients not receiving levothyroxine replacement; however, if the analysis was restricted to patients who were negative for thyroid autoantibodies, this association did not exist. These findings support the hypotheses mentioned above. Thyroid autoimmunity, which is common in morbid obesity, influences the thyroid secretory capacity that manifests as TSH elevation which eventually has an impact on weight management. Conversely, a mild elevation of TSH levels is also common in morbidly obese patients without thyroid autoimmunity (19). However, in patients without thyroid autoimmunity, it is not relevant to provide levothyroxine replacement since this medication does not lead to WL.

Vitamin D is a fat-soluble substance that is distributed into fat, muscle, liver tissues, and serum [8, 9]. Several observational studies have reported an association between lower 25(OH)D levels and higher fat mass [10, 22, 23]. Obese individuals may have lower 25(OH)D levels due to the sequestration of 25(OH)D in fat tissue and volumetric dilution [24]. The clinical importance of this product is expected to increase as the degree of obesity increases. However, interventional studies on 25(OH)D supplementation have reported contradictory results [25, 26]. In a recently conducted randomized controlled trial, vitamin D3 supplementation did not improve body fat percentage in participants with obesity or overweight at baseline, but it caused a slight improvement in body fat composition in normal-weight participants. In the vitamin D-supplemented group, visceral adipose tissue, truncal fat mass, and sarcopenia measurements were all improved in subjects who had total 25(OH)D median levels ≥97 nmol/L after 2 years of supplementation, compared with those who had total 25(OH)D below the median levels [26]. Another double-blind randomized controlled trial, including 218 postmenopausal overweight or obese women, observed that a vitamin D3 supplementation for 1 year during a WL program did not increase WL when compared with the placebo group. However, women who became 25(OH)D replete (levels ≥80 nmol/L), experienced greater improvements in body fat loss [25]. Our findings are consistent with those of both studies; patients with 25(OH)D level ≥ 75 nmol/L at the final visit had greater fat mass loss.

Compared with healthy individuals, patients with metabolic syndrome have lower phosphate levels [27]. Because fractional excretion of phosphate was similar in normal-weight, overweight, and obese individuals, it is assumed that the low phosphorus level observed in patients with metabolic syndrome can be attributed to a decreased dietary intake [27]. Phosphorus obtained from the diet has many important functions [12]. However, the industrialization and globalization of food markets has caused several changes in dietary habits. The dramatic increase in the consumption of refined cereals, oils, sugars, and sweeteners has caused a decrease in daily phosphorus ingestion [14, 28]. These changes have occurred in parallel with increase in obesity [29]. It has been hypothesized that low phosphorus levels may contribute to the development of obesity through its role in the regulation of food intake and thermogenesis. Additionally, phosphorus has the capacity for inducing physical activity [14]. Phosphorus supplementation in obese subjects in a weight-reducing program resulted in an increase in resting metabolic rate [30, 31]. Therefore, insufficient phosphorus levels may be a concern when evaluating obesity, especially morbid obesity.

Although the kidneys are the major regulators of phosphate homeostasis, serum phosphorus levels are also altered by intestinal phosphorus absorption that is mediated by sodium/phosphate cotransporters, which are regulated by both dietary phosphate intake and active vitamin D [12]. The role of these cotransporters in the maintenance of phosphate balance is most relevant during periods of low phosphate availability [32], during which the phosphorus intake and the availability of active vitamin D act together to restore phosphorus levels. In the present study, WL was achieved in the group that had sufficient levels of 25(OH)D and phosphorus and not in the group that had insufficient levels of 25(OH)D and phosphorus. These findings, in accordance with the previously mentioned studies, highlight the clinical importance of phosphorus availability and its determinants in patients with morbid obesity.

Some limitations of the study should be noted—first, this was a single-center, retrospective analysis of a systematically evaluated cohort. However, such analysis may omit the different approaches in obesity management which may result in the confounding of the investigated parameters. Second, the study cohort included only patients with T2DM and prediabetes. The results cannot be generalized to patients with healthy glucose metabolism. Third, the study cohort was euthyroid (TSH levels between 0.45 and 4.5 μIU/L) during follow-ups. However, our study cannot define the optimal cut-off levels of TSH, 25(OH)D, and phosphorus in this population. Fourth, the regression analysis revealed that approximately 50% of the total variation in WL% was explained by variance in the investigated independent variables. All patients were advised to adapt a healthier lifestyle, and although patients were not monitored, the remaining variance may be due to differences in dietary, exercise, and lifestyle habits. Lastly, the insulin resistance mechanism is one of the fundamental pathways involved in the pathophsiology of obesity. Considering a substantial proportion of the study population had established T2DM and had been receiving medications that may confound both insulin secretion and insulin sensitivity mechanisms, we find it more feasible not to include HOMA-IR, the most frequently used marker for insulin resistance in clinical practice, in the analysis, although it was available in some of the patients. Therefore, our study could not provide the answer for the probable mechanisms and the pathways interconnected to elucidate whether 25(OH)D and phosphorus are causal effectors. On the other hand, a double-blind randomized cross-over pilot study demonstrated that phosphorus supplementation recovers the blunted diet-induced thermogenesis (the increase in energy expenditure above the basal resting rate that occurs after the ingestion of food components and it constitutes 5–15% of total energy expenditure) in obese patients and enhances postprandial satiety, probably via restoring ATP (since its production is dependent on phosphorus availability) deficiency that is the prominent feature of insulin resistance [33]. Further interventional studies to investigate the probable mechanisms of action are warranted.

## Conclusion

In conclusion, our findings suggest that TSH, phosphorus, and 25(OH)D levels should be evaluated in patients with morbid obesity. TSH predictivity seems to be a function of thyroid autoimmunity present with increased frequency in this cohort. Thyroid hormone replacement may be considered in the presence of thyroid autoantibodies. In addition, lower phosphorus status along with insufficient 25(OH)D depots may be a barrier preventing WL in this population.

## Supplementary Information

**Additional file 1.**

## Data Availability

The data that support the findings of this study are available on request from the corresponding author.

## Abbreviations

25(OH)D: 25-hydroxyvitamin D
ATP: Adenosine triphosphate production
BFP: Body fat percentage
BMI: Body-mass index
fT3: Free triiodothyronine
fT4: Free thyroxine
HbA1C: Hemoglobin A1c
IQR: Interquartile range
PTH: Parathormone
SPINA-GD: The calculated sum activity of step-up deiodinases
SPINA-GT: The calculated thyroid’s secretory capacity
T2DM: Type 2 diabetes
TSH: Thyroid-stimulating hormone
VIF: Variance of inflation
WL: Weight loss
