# Supplementary material for: Serum TSH, 25(OH) D and phosphorus levels predict weight loss in individuals with diabetes/prediabetes and morbid obesity: a single-center retrospective cohort analysis
Source: BMC Endocr Disord. 2022 Nov 18;22:282. doi: 10.1186/s12902-022-01202-4 (PMC9673446; doi:10.1186/s12902-022-01202-4)
Supplement: Supplementary file 1 — Additional file 1. [file 12902_2022_1202_MOESM1_ESM.pdf]

Supplementary Table 1: Exclusion criteria

1. Follow-up duration <6 months,
2. Young age (<18 years old) or elderly (>75 years old) at the time of the first visit
3. TSH levels < 0.45 μIU/ml or ≥ 4.5 μIU/ml on any clinic visit,
4. Use of anti-thyroid medications,
5. Use of rapid-acting insulins or premixed insulins,
6. Use of antipsychotics,
7. Use of bisphosphonates,
8. Glucocorticoid use,
9. Antidementia medications,
10. Hypopituitarism/secondary hypothyroidism,
11. Uncontrolled diabetes mellitus (HbA1c ≥ 9.0 %) on any visit during the follow-up,
12. Normal glucose metabolism,
13. Acromegaly,
14. Adrenocortical insufficiency,
15. Endogenous Cushing's syndrome,
16. Active malignancy,
17. Chronic kidney disease (creatinine clearance <45 ml/minute),
18. Chronic liver disease,
19. Heart failure (New York Heart Association classification Class III and Class IV),
20. Primary hyperparathyroidism,
21. Hypoparathyroidism,
22. Hypophosphatemia (<2.5 mg/dL), or hyperphosphatemia (>4.5 mg/dL),
23. Organ transplantation,
24. Thyroidectomy,
25. Bariatric surgery,
26. Immobilization,
27. Pregnancy or lactation,
28. Severe compliance problems to medications.

Supplementary Table 2: Definitions of terms used in the text

Class II obesity: Body mass index is ≥ 35 and <40 kg/m^2

Class III obesity: Body mass index is ≥ 40 kg/m^2

Menopause: Permanent stop of menstrual periods for at least six months and elevated FSH (≥ 30 mIU/mL).

Follow-up duration: The duration between the first and last recorded visit, expressed in months

Frequently used medication: A drug that is used for at least in 5% of the study population and for at least 6 months

Hormone assays:

The quantitative analysis of the circulating substances was performed on an Architect c8000 (Abbott, Abbott Park, IL, USA) in the Clinical Biochemistry Department of Defne Hospital (Hatay, Turkey), using either enzymatic (glucose) or non-enzymatic (creatinine, calcium, phosphorus, albumin, transaminases) in vitro tests. The quantitative analysis of TSH, fT4, fT3, anti-thyroid peroxidase, anti-thyroglobulin, intact PTH levels was performed using a chemiluminescent microparticle immunoassay (Abbott, Abbott Park, IL, USA). The total 25(OH)D concentration was also measured. Total 25 (OH) D assays: The highest observed limit of blank value was 1.6 ng/mL, and the highest observed limit of detection value was 2.2 ng/ml. The highest observed limit of quantification value at ≤ 20% coefficient of variation was 2.4 ng/ml

Tanita-type BC-418 body composition analyzer: The Tanita-type BC-418 body composition analyzer provides estimated values for body fat ratio, body fat mass, and fat-free mass. Data were derived by the Dual Energy X-ray Absorptiometry method using bioelectrical impedance analysis via tetrapolar 8-point tactile electrodes (minimum and maximum weight capacity is 2 kg and 200 kg, respectively, percentage body fat range is 1%–75%, percentage body fat increments is 0.1%).

Statistical terms:

R^2: indicates how much of the total variation in the dependent variable was explained by the variance of the independent variables.

F-value: The p-value associated with the F-value determines whether the independent variables reliably predict the dependent variable.

Variance of inflation factor (VIF): was used to quantify the severity of multicollinearity.

Supplementary Table 3: Baseline characteristics of the study population (n=285) and their motivations to visit the endocrinology clinic

Age	51.3±11.1
Gender, Male	77 (27.0)
Female	208 (73.0)
PCOS	12 (5.8)
Menopause	120 (57.7)
Menopause duration (in years)	8.0 (4.0-14.8)
Smoking, No	229 (80.4)
Ex-smoker	13 (4.6)
Active smoker	43 (15.1)
Alcohol, No	253 (88.8)
Social drinker	17 (6.0)
Regular drinker	15 (5.3)
The main motivation to visit the Clinic (baseline),	
Diabetes management	112 (39.3)
Obesity, per se	95 (33.3)
Non-specific symptoms (fatigue, lassitude, decreased quality of life, etc.)	26 (9.1)
Nodular goiter evaluation	17 (6.0)
Thyroid hormone evaluation	11 (3.9)
Elevated PTH evaluation	9 (3.2)
Hypertension management	4 (1.4)
Menstrual irregularity evaluation	4 (1.4)
Hirsutismus evaluation	2 (0.7)
Erectile dysfunction	2 (0.7)
Adrenal incidentaloma evaluation	2 (0.7)
Hyperprolactinemia evaluation	1 (0.4)
The follow-up duration (in months)	22 (12-35)
Type 2 diabetes, at the first visit,	159 (55.8)
Prediabetes, at the first visit,	126 (44.2)
Number of visits to the polyclinic	4 (3-5)
Hypertension,	157 (55.1)

Dyslipidemia, on pharmacological treatment,	113 (39.6)

**Data are presented as mean±SD, median (IQR 25-IQR75) or n (%), as appropriate.*

Supplementary Table 4: Frequently used medications during the study period

	n (%)
Metformin	244 (85.6)
Acarbose	66 (23.2)
DPP-IV inhibitors	50 (17.5)
Exenatide	83 (29.1)
SGLT-2 inhibitors	54 (18.9)
Insulin secretegogues	44 (15.4)
Basal insulins	27 (9.5)
Orlistat	62 (21.8)
Antihypertensives	
ACEIs	28 (9.8)
ARBs	105 (36.8)
Diuretics	77 (27.0)
Dihydropyridine CCBs	41 (14.4)
Beta-blockers	68 (23.9)
Statins	104 (36.5)
Fluoxetine	63 (22.1)
Levothyroxine	109 (38.2)

ACEIs, angiotensin-converting-enzyme inhibitors; ARBs, angiotensin II receptor blockers; CCBs, calcium-channel blockers; DPP-IV inhibitors, dipeptidyl peptidase-4 inhibitors; SGLT-2 inhibitors: sodium-glucose

Supplementary Table 5: Spearman's correlations of thyroid hormone indices and 25(OH)D associations (measured at the last visit) with weight loss in the study population

	The entire group (n=285)		No LT4 replacement group (n=176)		Anti-thyroid antibody negative and no LT4 replacement group (n= 155)	
	R	p-value	R	p-value	R	p-value
TSH (μIU/L)	**-0.18**	**.002**	**-0.16**	**.032**	-0.09	.274
fT4 (pmol/L)	0.11	.076	0.05	.501	-0.07	.374
fT3 (pmol/L)	-0.08	.207	-0.04	.611	-0.03	.746
SPINA-GD (nmol/s)	-0.12	.051	-0.06	.437	0.02	.859
SPINA-GT (pmol/s)	-	-	**0.19**	**.018**	0.07	.405
25(OH)D (nmol/L)	**0.29**	**<.001**	**0.29**	**<.001**	**0.31**	**<.001**
Parathormone (pg/mL)	**-0.18**	**.003**	-0.14	.083	-0.16	.054
Calcium (mg/dL)	**0.16**	**.007**	0.16	**.038**	0.14	.090
Phosphorus (mg/dL)	**0.24**	**<.001**	**0.30**	**<.001**	**0.31**	**<.001**
